# Flexible Magnetic Polymer Composite Substrate with Ba_1.5_Sr_1.5_Z Hexaferrite Particles of VHF/Low UHF Patch Antennas for UAVs and Medical Implant Devices

**DOI:** 10.3390/ma13041021

**Published:** 2020-02-24

**Authors:** Sang-Eui Lee, Seong Pil Choi, Kyung-Sub Oh, Jaehwan Kim, Sang Min Lee, Kang Rae Cho

**Affiliations:** 1 Department of Mechanical Engineering, Inha University, Incheon 22212, Korea; surry1665@inha.edu (S.P.C.); jaehwan@inha.ac.kr (J.K.); 2 Smart Radar System, Rm 701, Innovalley A, 253, Pangyo-ro, Seongnam-si 13486, Gyeonggi-do, Korea; ksfaraday@msn.com; 3 Department of Electronic Engineering, Inha University, Incheon 22212, Korea; sanglee@inha.ac.kr; 4 Physical and Life Sciences Directorate, Lawrence Livermore National Laboratory, Livermore, CA 94550, USA

**Keywords:** antenna substrate, magnetodielectric composite, hexaferrite, VHF, UHF, UAV, MICS

## Abstract

Our goal is to fabricate flexible magnetic polymer composites as antenna substrates for very high frequency (VHF)/low ultra high frequency (UHF) antennas for unmanned aerial vehicles (UAVs) and medical devices. Magnetodielectric materials, which have permeability (*μ*) similar to permittivity (*ε*), have attracted great attention, because they facilitate miniaturization of microwave devices while keeping or enhancing electromagnetic characteristics. Mechanically millled Ba_1.5_Sr_1.5_Co_2_Fe_24_O_41_ (Ba_1.5_Sr_1.5_Z) hexaferrite particles were used to increase permeability in the interesting frequency band. The microwave properties of Ba_1.5_Sr_1.5_Z composites were predicted and measured. Hansen’s zero-order analysis of antenna bandwidth and electromagnetic field simulation showed that the hexaferrite-based flexible composite could enhance a bandwidth and achieve the miniaturization of antennas as well. The magnetic antenna substrates can be a good solution to integrate antennas into the UAVs whose dimensions are comparable to or larger than communication wavelength.

## 1. Introduction

Considerable efforts have been devoted to the miniaturization of high frequency devices to satisfy demands for electronic packaging and integration [1,2,3,4,5]. The antenna integration into manned aerial vehicles conflicts with the aerodynamic efficiency of their structures, and interests in overcoming the confliction have recently increased by the advent of the so-called era of unmanned aerial vehicles (UAVs) [6,7,8,9,10,11,12,13]. The communication and data link with the UAVs cover frequency bands from high frequency (HF) band to Ku band, 3 MHz–18 GHz [6]. The antenna integration strongly depends on the size of the UAVs, especially, for low frequency bands like HF and Very High Frequency (VHF), due to the corresponding wavelength.

In order to incorporate antennas into electronic devices with limited space, high dielectric and low loss materials have been used as antenna substrates [4,5]. This approach has been adopted in the development of conformal load-bearing antenna structure (CLAS) for aerial vehicles including UAVs, which use microwave frequency ranges including VHF and Ultra High Frequency (UHF) [10,11,12,13], as well as implantable antennas for short-distance biotelemetry for the Medical Implant Communication Service (MICS) band, 402–405 MHz [14,15,16,17]. However, the use of dielectric-only substrates has drawbacks; field concentration around the high permittivity region leads to narrow bandwidth and low antenna efficiency, and low characteristic impedance, causing difficulties in impedance matching [1].

The use of magnetodielectric (MD) materials or magnetic materials with relative real permeability higher than one (*μ_r_′* > 1) makes it possible to overcome such drawbacks [1,2,18]. While a variety of magnetic materials such as spinel ferrites like MFe_2_O_4_ (M is a metallic ion with two valence electrons, 2+) have the magnetic resonance frequencies below 100 MHz [19,20], there are also hexagonal ferrites (or hexaferrites) whose magnetic resonance frequencies exceed Snoek’s limit for an isotropic material [19]. The structural types of the hexaferrites determine the magnetic properties and related microwave absorption/transmission [21], as well as multiferroics [22]. Ba_3_Z (Ba_3_Co_2_Fe_24_O_41_) Z-type hexaferrite ceramic with *μ_r_′* = *ε_r_′* (real permittivity) = 16 was successfully demonstrated for substrates of an antenna operating at 277 MHz [1]. Another Z-type hexaferrite, Ba_1.5_Sr_1.5_Co_2_Fe_24_O_41_ (Ba_1.5_Sr_1.5_Z) bulk ceramic material was developed by substituting Ba^2+^ with Sr^2+^, and it was observed to have a higher magnetic resonance frequency and lower magnetic loss than Ba_3_Co_2_Fe_24_O_41_ (Ba_3_Z) near 400 MHz frequency range [23]. However, the oxide structures are vulnerable to fracture under mechanical loading conditions, and thus flexible substrates can be preferable to the ferrimagnetic oxides for their application to the load bearing antennas [10,11,12,13] as well as RFID (Radio-Frequency Identification) antennas [2,24,25].

In this study, magnetic composites containing Ba_1.5_Sr_1.5_Z particles in a flexible polymer were fabricated to investigate feasibility of antenna substrate material operating at VHF/low UHF ranges for its application to aerial vehicle antennas. The microwave properties of the composites were characterized and evaluated in comparison with electromagnetic mixing formulas. The characteristics of the hexaferrite composites as patch antenna substrate were examined by Hansen’s zero-order analysis and electromagnetic field simulation in the viewpoint of miniaturization and antenna performance.

## 2. Experimental

### 2.1. Materials

The powder and bulk ceramic of Ba_1.5_Sr_1.5_Z were synthesized by a solid-state ceramic process from starting materials of BaCO_3_ (99.7%) SrCO_3_ (99.7%), Co_3_O_4_ (99.9%) and Fe_2_O_3_ (99.5%). The weighed mixture of the precursors was homogenized by zirconia balls in ethanol for 24 h, and calcined in an alumina crucible at 1000 °C in air for 4 h. Then the calcined powder samples were crushed by hand grinding and palletized with a pressure of 7.5 MPa. The pallets were annealed in air at 1250 °C for 16 h. Then, the sintered ceramics were mechanically ground into powders by a high pressure grinding mill. After sieving with an ASTM no. 140 sieve (opening: 106 μm), the Ba_1.5_Sr_1.5_Z powder of a length less than the opening size was used in a polymer matrix. The polyurethane (PU, CAAP Co., Milford, CT, USA) system used as matrix consists of three parts; urethane monomers consisting of 80 wt % diisocyanate and 20 wt % diol, a catalyst containing aliphatic amine, parachlorobenzotrifluoride and methyl propylketone and the accelerator containing 1 wt % organotitanate and 99 wt % acetone (polyurethane STD-102).

### 2.2. Composite Fabrication

The fabrication of the Ba_1.5_Sr_1.5_Z/PU composite starts with mixing the Ba_1.5_Sr_1.5_Z powder with the urethane monomer. Then, the mixture was sonicated for 2 h and stabilized in cold water during ultrasonication for an additional one hour. The catalyst and the accelerator were introduced into the aforementioned mixture and the suspended solution was ultrasonically stirred for 5 min and then poured into a mold for curing and solvent evaporation. Post curing was made at 100 °C for 2 h.

### 2.3. Characterization

Micrographs of particles and composites were observed by a scanning electron microscope (SEM, JEOL SM-71010, Tokyo, Japan). The Ba_1.5_Sr_1.5_Z powders were characterized using X-ray diffraction (XRD, X’PERT MPD, Philips) with a Cu K_α_ radiation source (k = 0.154056 nm). The measurement of electromagnetic properties was carried out with an Agilent E4991A (Santa Clara, California, USA) impedance analyzer, combined with an Agilent 16453A dielectric material test fixture and an Agilent 16454A magnetic material test fixture in the frequency range, 0.01–1.0 GHz. Specimens for permittivity measurement were cylinders with a diameter of 17.5 mm, and those for permeability were hollow cylinders with an inner diameter of 10 mm and an outer diameter of 17.5 mm. The thicknesses of all the samples ranged from 0.5 to 2.0 mm. Antenna performance, radiation pattern and return loss of an antenna with composite substrate were simulated by using FDTD (finite difference time domain) approach.

## 3. Results and Discussion

### 3.1. Materials and Electromagnetic Property

**Figure 1:**
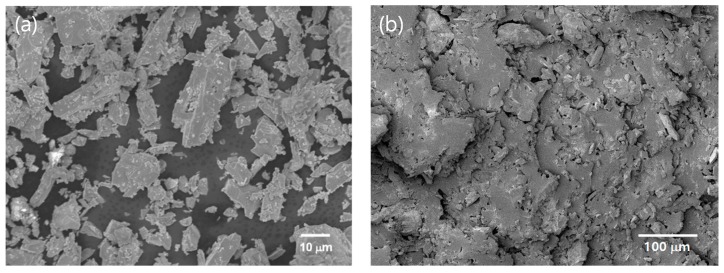
SEM images of (**a**) Ba_1.5_Sr_1.5_Z particles, and (**b**) 21.4 Vol % Ba_1.5_Sr_1.5_Z/PU composite.

**Figure 2:**
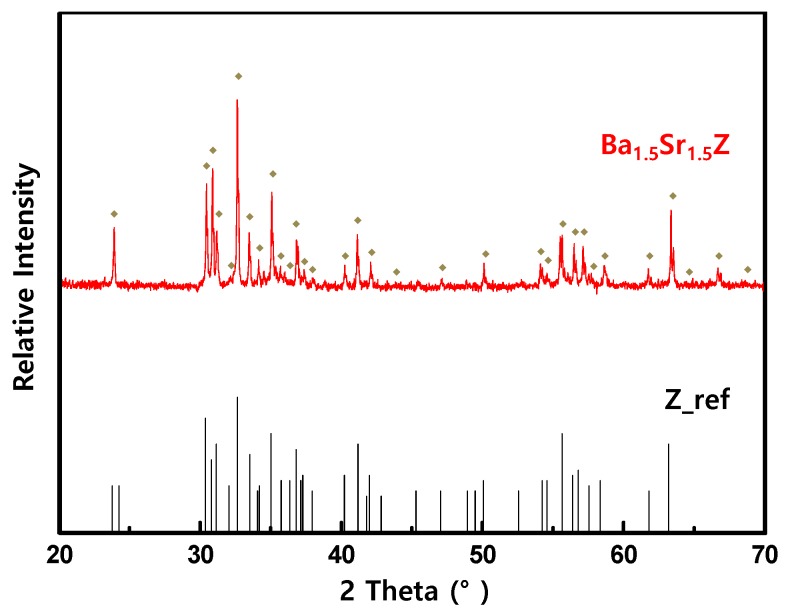
X-ray diffraction pattern of the Ba_1.5_Sr_1.5_Z powder.

As shown in Figure 1, Ba_1.5_Sr_1.5_Z particles have irregular shapes including flake-like and needle-like particles. Grain sizes of the particles are observed in micro-size level due to the high temperature sintering at 1250 °C [23]. Figure 1b shows the fractured surface of the Ba_1.5_Sr_1.5_Z (21.4 Vol %)/PU. The particles were randomly and evenly dispersed in the matrix. Figure 2 shows XRD pattern of the synthesized powders. The diffraction peaks for the hexaferrite powder are indexed and compared with a reference data. The particles are observed to consist of almost the Z-type hexaferrite [23,26].

**Figure 3:**
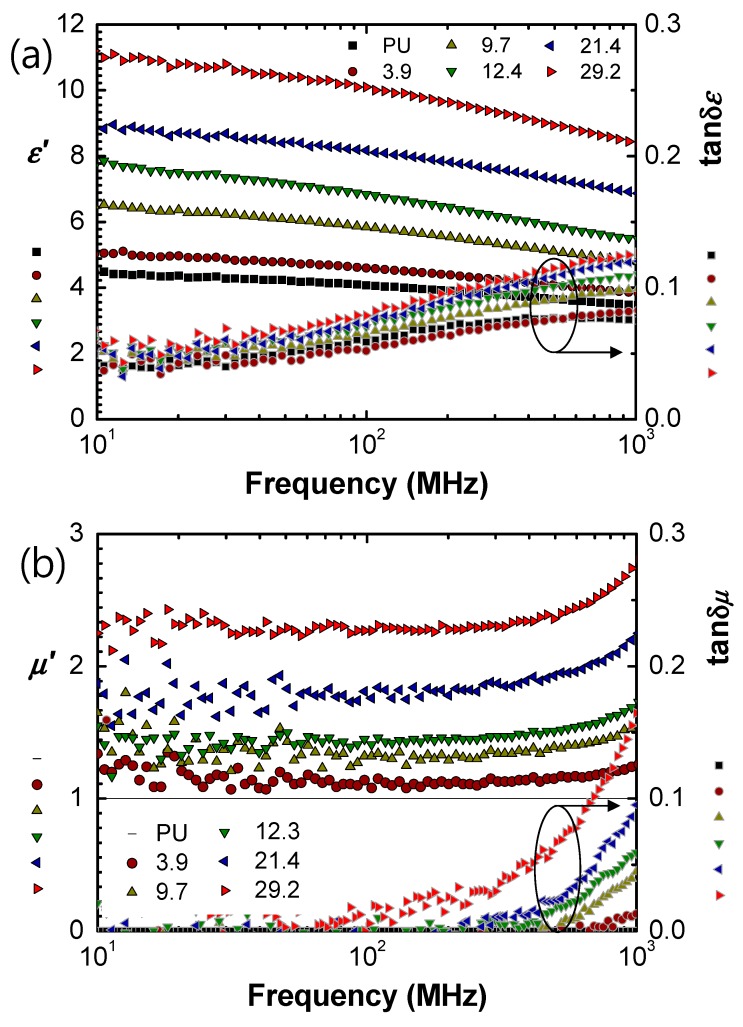
(**a**) Permittivity and (**b**) permeability of Ba_1.5_Sr_1.5_Z/PU.

**Figure 4:**
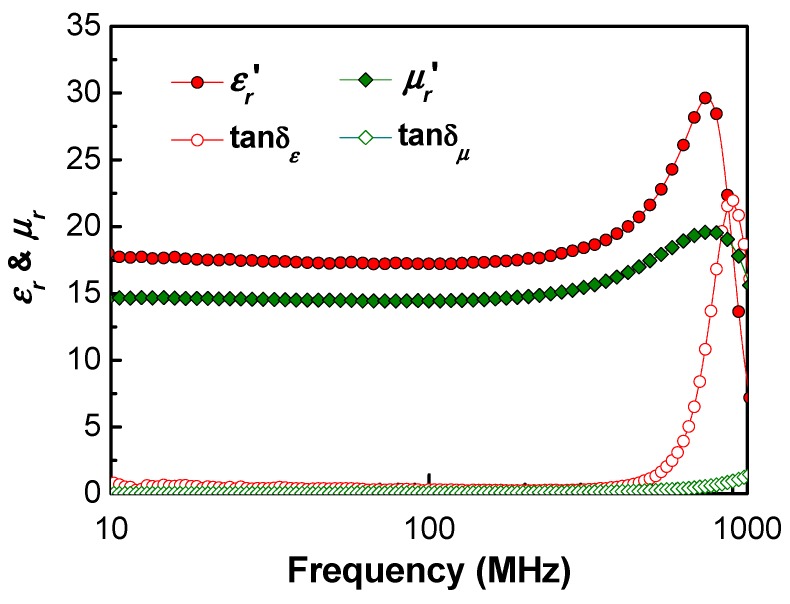
Permittivity and permeability of bulk Ba_1.5_Sr_1.5_Z.

Figure 3 shows the permittivity and permeability for the Ba_1.5_Sr_1.5_Z/PU. Both properties increased, scaling with the volume fraction of Ba_1.5_Sr_1.5_Z in the composites. The real permittivity decreased a little with increasing frequency, while the resonant frequencies existed from 300 to 500 MHz. The real permeability was kept in a constant level as the frequency increased near 400 MHz. Magnetic resonance frequency was not observed in the interesting frequency range up to 1 GHz, because the resonance frequency of the hexagonal ferrite is more than 2–3 GHz [1,19,27]. In addition, the magnetic resonance frequency of polymer composites with magnetic particles was observed to be higher than that of the bulk ceramics [27,28], which is also observed from the comparison of Figure 3 with Figure 4.

Dielectric loss (tan *δ_ε_*) of the MD composites was observed to be relatively high over 0.05. By adopting polymers with low loss, such as polydimethylsiloxane (PDMS) [29], or polycarbonate (PC) [30] as a host matrix, the dielectric loss is expected to be further optimized and reduced. Magnetic loss (tan *δ_μ_*) increased as the filler fraction increased, especially above 400 MHz. Therefore, it can be concluded that Ba_1.5_Sr_1.5_Z/PU in *V_f_* < 21.4% can be a good candidate for antenna substrate in a few hundred MHz range.

The material properties of the bulk Ba_1.5_Sr_1.5_Z were also measured for input data of Equations (1)–(3). Its real permittivity and permeability were measured to be 17.4 and 14.5 at 402 MHz, respectively. The permeability was a little bit lower than 17.0 of Ba_1.5_Sr_1.5_Z with the same composition [23]. However, the bulk oxide in that study had the resonance frequency higher than 1 GHz and the value of around 1 GHz [23]. The difference might be attributed to sintering condition like temperature and oxygen partial pressure [23].

The microwave properties of the composite can be calculated by following Bruggeman equation (BG) and quasi-crystalline approximation with coherent potential (QCA-CP) [30,31], using the measured microwave properties of the bulk hexaferrite in Figure 4.

(1) $$\frac{P_{c}-P_{m}}{P_{c}+P_{m}+\nu \left(P_{c}-P_{m}\right)}=V_{f}\frac{P_{f}-P_{m}}{P_{f}+P_{m}+\nu \left(P_{c}-P_{m}\right)}$$

where *P* means the permittivity (*ε_r_*,) or permeability (*μ_r_*), *V_f_* is the particle volume fraction and the subscripts, *c*, *f*, and *m*, denote composite, filler, and matrix. *υ* is assigned 2 for BG and 3 for QCA-CP.

The material properties are also reported to be bound with Wiener’s upper and lower bounds [30]. The upper bound is also known as Lichtenecker’s equation [31,32].

(2) $$P_{c}=V_{f}P_{c}+\left(1-V_{f}\right)P_{m}$$

(3) $$\frac{1}{P_{c}}=\frac{V_{f}}{P_{f}}+\frac{1-V_{f}}{P_{m}}$$

**Figure 5:**
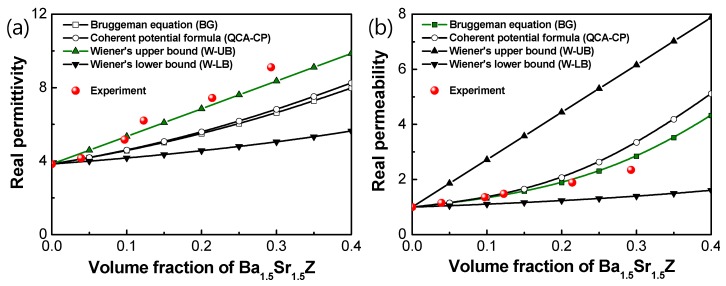
(**a**) Real permittivity and (**b**) real permeability of Ba_1.5_Sr_1.5_Z/PU with filler volume fraction.

The experimental data of the real permittivity of the composites were found in good agreement with Wiener’s upper bound, as shown in Figure 5a. The experimental results were higher than those predicted by BG and QCA-CP. This underestimation of the dielectric constant by the above Equations (2) and (3) can be believed to be due to the formation of large particle clusters at high particle loading [24] and also irregular particle shapes like needle or flake types, which may lead to higher real permittivity in comparison with spherical particles [32].

The measured real permeability of the composites was in good agreement with the data calculated by BG, as shown in Figure 5b. The dependence of the real permittivity and permeability on the filler volume fraction were consistent with those for Ba_3_Z particle/PVDF (polyvinylidene fluoride) composites [28].

### 3.2. Antenna Performance

**Figure 6:**
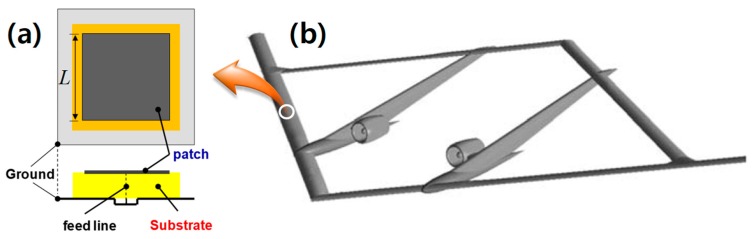
(**a**) Schematic of a planar antenna with a substrate onto (**b**) a concept sensor-craft [33].

Figure 6 shows a planar patch antenna whose performance depends on material properties of its substrate, as well as geometrical features including substrate thickness and patch size [1]. In general, development of skin antennas requires concurrent consideration of structural integration in addition to the antenna performance [10]. Antenna performance with the magnetodielectric composite substrates was investigated and compared with that of an antenna having a pure dielectric (PD) substrate with the same miniaturization factor, (*μ_r_′*·*ε_r_′*)^1/2^, that is, (*μ_r_′*·*ε_r_′*)_MD_ = (*ε_r_′*)_PD_.

For the comparison between them, the impedance, the miniaturization factor and the bandwidth of the polymer composite substrates were calculated. Zero-order antenna bandwidth proposed by Hansen [2] can be evaluated by Equation (4).

(4) $$BW=\frac{96\sqrt{\mu_{r}^{\prime }/\epsilon_{r}^{\prime }}\left(t/\lambda_{0}\right)}{\sqrt{2}\left(4+17\sqrt{\mu_{r}^{\prime }/\epsilon_{r}^{\prime }}\right)}$$

where *BW* is the bandwidth, *t* is the thickness of an antenna substrate and *λ*_0_ is the wavelength in free space at resonance frequency. *μ_r_′* and *ε_r_′* are real permeability and permittivity, respectively.

Table 1 summarizes miniaturization factors and impedances of the fabricated composites at 402 MHz. The improvement in the bandwidth of a composite material with both non-unit *μ_r_′* and *ε_r_′* can be evaluated by comparing that of a PD material, which has the same miniaturization factor ((1.5 × 6.2)^1/2^ = 3.1) as 12.3 Vol % MD composite. In the table, *BW* and *BW_ε_* are bandwidths of MD and PD materials, respectively and *BW*/*BW_ε_* is calculated to be equal to *μ_r_′* by Equation (4). In addition, the miniaturization factor increases by the scale of *μ_r_′*. Comparison of 12.3 Vol % MD composite with the corresponding PD material as antenna substrate implies that MDs can have the same miniaturization factors, while they can have higher relative impedance, (*μ_r_′*/*ε_r_′*)^1/2^, closer to that of free space (=1.0), and also higher bandwidths.

**Figure 7:**
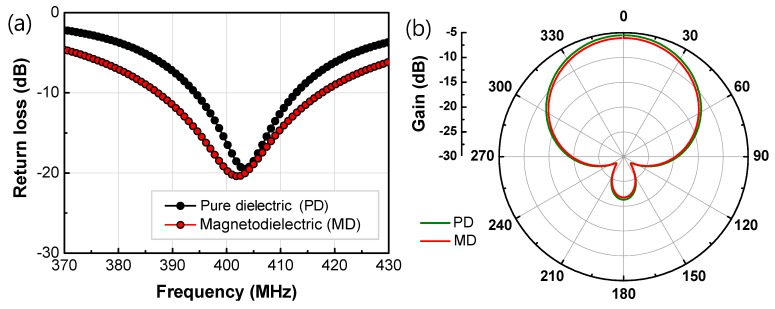
(**a**) Return loss and (**b**) radiation pattern for antennas of 12.3 Vol % MD and the corresponding PD materials.

Figure 7 shows return loss and radiation pattern for antennas with 12.3 Vol % MD composite, and the corresponding PD material by simulation, respectively. The geometry and microwave performance of the simulated patch antennas in Figure 7 are summarized in Table 2.

With optimization of feeding points of the antennas, parameters were calculated. The optimized results showed that gain (*G*) and radiation efficiency (*η*_rad_) might be slightly degenerated, but *BW* was greatly enhanced by around 63%, which is consistent with Hansen’s prediction of 50% *BW* improvement by Equation (4). The difference can be attributed to dielectric and magnetic losses in the real materials, as Equation (4) is derived with assumption of no loss.

## 4. Conclusions

We fabricated Ba_1.5_Sr_1.5_Z particle/PU polymer composites for substrate of antennas for UAVs in VHF and low UHF frequency ranges. The synthesized particles were Z-type hexaferrite as proved by the XRD analysis. The permittivity and permeability of the composites were evaluated to characterize their antenna performance in terms of miniaturization factor and bandwidth. The real permittivity of the Ba_1.5_Sr_1.5_Z particle/PU composites obeyed Wiener’s upper bound, while the real permeability agreed with Bruggeman equation. It was observed from the measured dielectric and magnetic loss that Ba_1.5_Sr_1.5_Z/PU composites with *V_f_* < 21.4% can be a good candidate for antenna substrate in a few hundred MHz range.

The antenna performance simulation showed that the magnetodielectric Ba_1.5_Sr_1.5_Z particle/PU polymer composites had advantages over pure dielectric materials in the viewpoint of bandwidth. It was observed that the hexaferrite composites possessed higher bandwidth (62.9% enhanced) and smaller antenna area (9.0% reduced), compared with the dielectric-only material.

In addition, microwave properties of the composites are expected to be further optimized by controlling dielectric and magnetic losses of particles and polymer, because the polyurethane used in this study was with relatively high dielectric loss, compared with a general or low-loss dielectric polymer. The magnetodielectric materials with Z-type hexaferrite incorporated into a polymer can extend the design space of planar antennas.

## Figures and Tables

**Table 1:** Microwave characteristic of magnetodielectrics (MDs) and pure dielectric (PD).

	*V_f_*	*μ_r_′*	*ε_r_′*	(*μ_r_′ε_r_′*)^1/2^	(*μ_r_′*/*ε_r_′*)^1/2^	*BW*/*BW*_ε_ *
MD	03.9	1.2	4.2	2.2	0.53	1.2
	09.7	1.4	5.2	2.7	0.52	1.4
	12.3	1.5	6.2	3.1	0.49	1.5
	21.4	1.9	7.4	3.7	0.51	1.9
	29.2	2.4	9.1	4.6	0.51	2.4
PD	-	1	9.3	3.1	0.33	-

* *BW*/*BW_ε_* (*=μ_r_′*) is calculated on the basis of Equation (4).

**Table 2:** Geometry and characteristic of patch antennas with 12.3 Vol % MD composite and with PD material as a substrate.

Parameter (*P*)	Unit	MD	PD	Δ *
*L* (length)	cm	12.5	13.1	−4.6 (%)
*A* (area)	cm^2^	156.3	171.6	−9.0 (%)
G (gain)	dB	−6.1	−5.5	−11.0 (dB%)
*η*_rad_	dB	−12.8	−12.3	−4.1 (dB%)
BW	dB	30.3	18.6	62.9 (%)

* Δ(%) is calculated on (*P*|_MD_ − *P*|_PD_)/*P*_PD_ × 100.
